# Population-based estimates of breast cancer risk for carriers of pathogenic variants identified by gene-panel testing

**DOI:** 10.1038/s41523-021-00360-3

**Published:** 2021-12-09

**Authors:** Melissa C. Southey, James G. Dowty, Moeen Riaz, Jason A. Steen, Anne-Laure Renault, Katherine Tucker, Judy Kirk, Paul James, Ingrid Winship, Nicholas Pachter, Nicola Poplawski, Scott Grist, Daniel J. Park, Bernard J. Pope, Khalid Mahmood, Fleur Hammet, Maryam Mahmoodi, Helen Tsimiklis, Derrick Theys, Amanda Rewse, Amanda Willis, April Morrow, Catherine Speechly, Rebecca Harris, Robert Sebra, Eric Schadt, Paul Lacaze, John J. McNeil, Graham G. Giles, Roger L. Milne, John L. Hopper, Tú Nguyen-Dumont

**Affiliations:** 1 Precision Medicine, School of Clinical Sciences at Monash Health, Monash University, Melbourne, Australia; 2 Department of Clinical Pathology, The University of Melbourne, Melbourne, Australia; 3 Cancer Epidemiology Division, Cancer Council Victoria, Melbourne, Australia; 4 Centre for Epidemiology and Biostatistics, The University of Melbourne, Melbourne, Australia; 5 Department of Epidemiology and Preventive Medicine, School of Public Health and Preventive Medicine, Monash University, Melbourne, Australia; 6 Hereditary Cancer Centre, Nelune Comprehensive Cancer Centre, Prince of Wales Hospital, Sydney, Australia; 7 Familial Cancer Services, Westmead Hospital, Westmead, Australia; 8 Peter MacCallum Cancer Centre, Melbourne, Australia; 9 Royal Melbourne Hospital, Melbourne, Australia; 10 Present Address: Department of Medicine, The University of Melbourne, Melbourne, Australia; 11 King Edward Memorial Hospital, Perth, Australia; 12 Adult Genetics Unit, Royal Adelaide Hospital, Adelaide, Australia; 13 School of Medicine, University of Adelaide, Adelaide, Australia; 14 SA Pathology, Flinders Medical Centre, Adelaide, Australia; 15 Melbourne Bioinformatics, The University of Melbourne, Melbourne, Australia; 16 Department of Biochemistry and Molecular Biology, The University of Melbourne, Melbourne, Australia; 17 Department of Genetics and Genomic Sciences, Icahn School of Medicine at Mount Sinai, New York, NY USA

**Keywords:** Breast cancer, Genetic testing

## Abstract

Population-based estimates of breast cancer risk for carriers of pathogenic variants identified by gene-panel testing are urgently required. Most prior research has been based on women selected for high-risk features and more data is needed to make inference about breast cancer risk for women unselected for family history, an important consideration of population screening. We tested 1464 women diagnosed with breast cancer and 862 age-matched controls participating in the Australian Breast Cancer Family Study (ABCFS), and 6549 healthy, older Australian women enroled in the ASPirin in Reducing Events in the Elderly (ASPREE) study for rare germline variants using a 24-gene-panel. Odds ratios (ORs) were estimated using unconditional logistic regression adjusted for age and other potential confounders. We identified pathogenic variants in 11.1% of the ABCFS cases, 3.7% of the ABCFS controls and 2.2% of the ASPREE (control) participants. The estimated breast cancer OR [95% confidence interval] was 5.3 [2.1–16.2] for *BRCA1*, 4.0 [1.9–9.1] for *BRCA2*, 3.4 [1.4–8.4] for *ATM* and 4.3 [1.0–17.0] for *PALB2*. Our findings provide a population-based perspective to gene-panel testing for breast cancer predisposition and opportunities to improve predictors for identifying women who carry pathogenic variants in breast cancer predisposition genes.

## Introduction

Data from breast cancer predisposition gene panel testing are accumulating rapidly as it becomes more affordable and more accessible in different settings, including clinical care. Large studies based in clinical and commercial testing laboratories have demonstrated that gene panel testing has increased the number of clinically actionable variants identified in women undergoing testing, compared with previous testing that included only *BRCA1* and *BRCA2*. This increase in actionable findings is in the order of 5–10% depending on the setting, inclusion criteria for actionable variants and study design^1–5^.

Most of this work has been based on women selected for high-risk features, such as personal or family history of breast cancer, who underwent gene panel testing for cancer susceptibility at commercial laboratories^6–8^. Far fewer data are available to make inference about breast cancer risk for women unselected for family history, which is important to consider for population screening of affected and unaffected women. The value of population-based case-control studies and gene panel testing have recently been illustrated by Hu et al.^9^ who reported the outcome of a US-based study (CARRIERS consortium), involving over 32,000 affected and 32,000 unaffected women and Dorling et al.^10^ who reported the outcome of an international study (BRIDGES) involving 60,000 women affected and over 53,000 women unaffected by breast cancer. These studies provided improved estimates of the prevalence and the magnitude of breast cancer risk associated with pathogenic variants in known breast cancer predisposition genes to guide genetic counselling.

Several studies have only included women affected by breast cancer (case only) and have reported variant prevalence^1–4^. Kurian et al.^11^ linked cancer registries from Georgia and California (USA) to the gene panel testing outcomes from four key clinical testing laboratories. Their study linked 24.1% of the 77,085 women with breast cancer to genetic testing results and reported that panel testing increased the frequency of actionable genetic findings by 1.5%. Several large studies have reported estimates of breast cancer risk associated with carrying a rare germline variant in a gene included in these gene panel tests^7–10,12–16^.

Here, we report the prevalence and breast cancer risk estimates associated with pathogenic rare variants identified in breast cancer predisposition gene panel tests, conducted in an Australian population-based case-control study of breast cancer (with an emphasis on early age at disease onset), involving both (i) age-matched population-based controls and (ii) a healthy older group of Australian women as controls.

## Results

### Study subjects

**Fig. 1:**
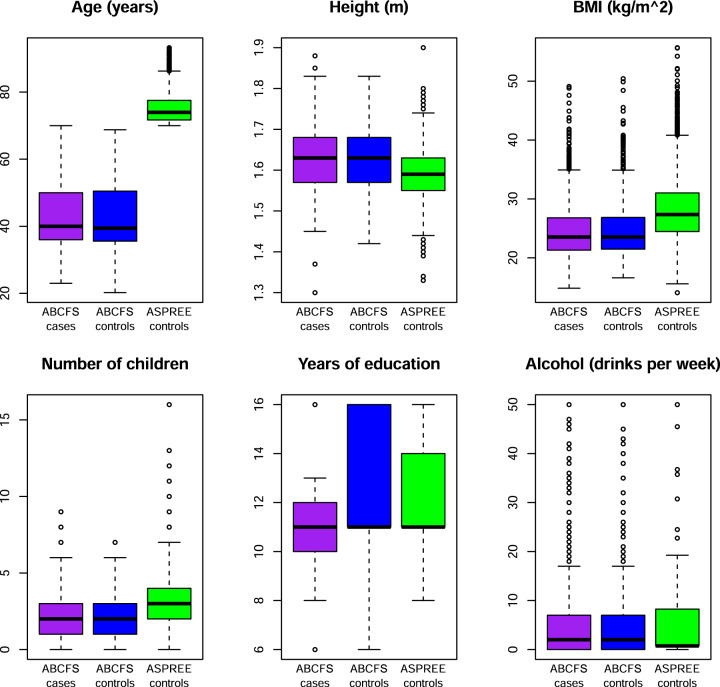
Box-and-whisker plots of potential confounders. Boxplots of potential confounders, used as adjustment variables in the analyses, for controls from the ASPirin in Reducing Events in the Elderly (ASPREE; in green) study, and for cases and controls from the Australian Breast Cancer Family Study (ABCFS; in purple and blue). Panels are for age (years), height (m), body mass index (kg/m^2^), number of children, education (years) and alcohol consumption (drinks per week), as indicated. For each boxplot, the horizontal lines are at the potential confounder’s median (bold line), 25th and 75th percentiles (horizontal bounds of the box) and most extreme data points within a distance, from the box, of 1.5 times the interquartile range (shorter horizontal lines).

Table 1 and Fig. 1 give descriptive statistics for the study subjects. Regarding the six potential confounders that we used as adjustment variables in our main analyses, the ABCFS cases and controls were very similar to each other, and both were similar to the ASPREE controls except that the ASPREE controls were older, as expected due to study design differences.

**Table 1:** Descriptive statistics for the subjects participating in the ASPirin in Reducing Events in the Elderly (ASPREE) study and the Australian Breast Cancer Family Study (ABCFS) included in this study, by breast cancer affected status.

	ASPREE controls	ABCFS cases	ABCFS controls
Number of subjects	6549^b^	1464	862
Cases, number (%)	0 (0%)	1464 (100%)	0 (0%)
Controls, number (%)	6549 (100%)	0 (0%)	862 (100%)
Carriers of a pathogenic^a^ variant in any gene, number (%)	145 (2%)	162 (11%)	32 (4%)
Median (IQR) age in years at diagnosis (for carriers) or at baseline (for controls)	74 (5.8)	40 (14)	39.4 (14.8)
Height in m, median (IQR)	1.59 (0.08)	1.63 (0.11)	1.63 (0.11)
Body mass index in kg/m^2^, median (IQR)	27.4 (6.6)	23.5 (5.5)	23.5 (5.4)
Number of children, median (IQR)	3 (2)	2 (2)	2 (2)
Years of education, median (IQR)	11 (3)	11 (2)	11 (5)
Alcoholic drinks per week, median (IQR)	1 (8)	2 (7)	2 (7)

*IQR* inter-quartile range, kg kilograms, m metres.

^a^Pathogenic (including likely pathogenic) as defined by ClinVar and protein-truncating variants that are absent from ClinVar (accessed July 2020). Excludes carriers of protein-truncating variants located in the last coding exon and mono-allelic carriers of a MUTYH variant.

^b^Female participants to ASPREE for whom genetic data were available, excluding women with a prior diagnosis of breast cancer and carriers of more than one pathogenic variant.

### Gene panel testing

There were 162 (11%) ABCFS cases with a pathogenic variant, compared with 32 (4%) of the ABCFS controls and 145 (2%) of the ASPREE controls (Table 2). Further details of pathogenic variants detected are provided in Supplementary Tables 1–3 for the ABCFS cases, ABCFS controls and ASPREE participants, respectively. The number of carriers of pathogenic variants in genes other than *BRCA1* or *BRCA2* in the ABCFS cases, ABCFS controls and ASPREE controls were 73 (5%), 22 (3%) and 128 (2%) respectively.

**Table 2:** Pathogenic variant carriers identified by gene-panel testing and the odds ratios (ORs) and corresponding 95% confidence intervals (CIs) for associations with breast cancer.

	Cases (*n* = 1464)		Controls (*n* = 7411)			
Gene	Number of carriers	%	Number of carriers	%	Adjusted^a^ OR (95% CI)	*p*-value
*ATM*	17	1.2%	25	0.3%	3.4 (1.4–8.4)	0.0085
*BARD1*	3	0.2%	3	0%	8.2 (0.73–83)	0.09
*BRCA1*	46	3.1%	6	0.1%	5.3 (2.1–16.2)	0.00011
*BRCA2*	43	2.9%	21	0.3%	4 (1.9–9.1)	0.00016
*BRIP1*	8	0.5%	13	0.2%	2.8 (0.77–9.9)	0.12
*CDH1*	1	0.1%	0	0%	–	–
*CHEK2*	19	1.3%	35	0.5%	1.3 (0.53–3)	0.61
*FANCM*	3	0.2%	19	0.3%	0.8 (0.12–4.2)	0.81
*MLH1*	0	0%	0	0%	–	–
*MRE11A*	0	0%	5	0.1%	–	–
*MSH2*	0	0%	1	0%	–	–
*MSH6*	3	0.2%	3	0%	4.7 (0.52–41.3)	0.16
*MUTYH*^b^	0	0%	0	0%	–	–
*NBN*	1	0.1%	11	0.1%	2 (0.09–14.3)	0.6
*NF1*	2	0.1%	4	0.1%	4.7 (0.4–40)	0.21
*PALB2*	7	0.5%	10	0.1%	4.3 (1–17)	0.043
*PMS2*	0	0%	0	0%	–	–
*PTEN*	0	0%	1	0%	–	–
*RAD50*	2	0.1%	11	0.1%	0.3 (0.04–2)	0.22
*RAD51C*	0	0%	4	0.1%	–	–
*RAD51D*	1	0.1%	4	0.1%	0.25 (0.01–5.5)	0.35
*STK11*	0	0%	0	0%	–	–
*TP53*	6	0.4%	1	0%	19.9 (0.9–1125)	0.062

Pathogenic (including likely pathogenic) as defined by ClinVar and protein-truncating variants that are absent from ClinVar (accessed July 2020). Excludes carriers of protein-truncating variants located in the last coding exon.

^a^Adjusted for age, height, body mass index, number of children, number of years of education and number of alcoholic drinks per week.

^b^This study excludes mono-allelic carriers of a *MUTYH* pathogenic variant, of which there were 13 and 47 carriers among case subjects and control subjects, respectively.

### Statistical analyses

**Fig. 2:**
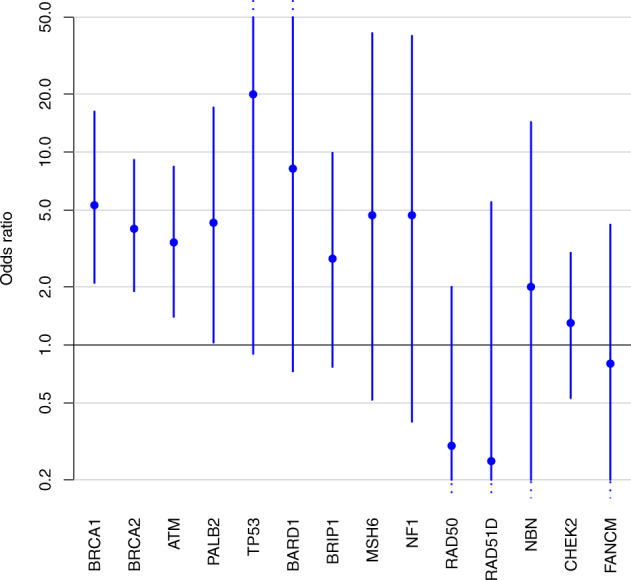
Pathogenic variants and breast cancer risk. Adjusted odds ratios (large dots) and corresponding 95% confidence intervals (vertical lines) for the association between breast cancer and pathogenic variants in various genes, sorted by *p*-value.

We found evidence of association with breast cancer risk for four genes, with estimated adjusted ORs of 5.3 [95% CI: 2.1–16.2] for *BRCA1*, 4.0 [95% CI: 1.9–9.1] for *BRCA2*, 3.4 [95% CI: 1.4–8.4] for *ATM* and 4.3 [95% CI: 1.0–17.0] for *PALB2* (Table 2, Fig. 2). *CHEK2* was the gene that carried the highest number of pathogenic variants after *BRCA1* and *BRCA2*. We observed no evidence of an association between pathogenic variants in this gene and breast cancer risk in our main analyses (OR 1.3 [95% CI: 0.53–3, *p* = 0.6]), though there was evidence of an association from unadjusted analyses (*p* = 0.0009; Supplementary Table 4), which give biased estimates of risk but valid tests of association. Our study also found no statistical difference between c.1100delC (OR 1.1 [95% CI: 0.29–3.8]) and all other CHEK2 pathogenic variants (OR 1.4 [95% CI: 0.44–5.1]), *p* = 0.75. For the other genes included in our analysis, the evidence for an association between breast cancer and pathogenic variants was weak or absent, and did not reach statistical significance (Table 2). Adjustment for age had a large influence on the ORs for some genes, as expected, but further adjustment for the remaining potential confounders had a relatively small effect (Supplementary Table 4).

Breast cancer case subjects with pathogenic variants in *BRCA1* or *BRCA2* had, on average, a younger age at diagnosis than case subjects with pathogenic variants in genes other than *BRCA1* or *BRCA2* (*p* = 0.002), with average ages at diagnosis of 38.2 [95% CI: 36.7–39.8] years and 42.4 [95% CI: 40.3–44.6] years, respectively. Carriers with pathogenic variants in *BRCA1* or *BRCA2* were also more likely to have a family history of breast cancer than carriers of pathogenic variants in the other genes (*p* < 0.0001).

Pathogenic variants in *BRCA1* were strongly associated with an increased risk of ER-negative breast cancer (Supplementary Table 5). There was also weak evidence that pathogenic variants in *CHEK2* were associated with risk of ER-negative breast cancer, though only after age adjustment. Pathogenic variants in *ATM*, *BRCA1*, *BRCA2* and *PALB2* were all associated with the risk of ER-positive breast cancer (Supplementary Table 6). Carriers of pathogenic variants in *BRCA1* were less likely than non-carriers to have ER-positive breast cancer, presumably as a consequence of the strong association between pathogenic variants in *BRCA1* and ER-negative breast cancer, since ER-positive and ER-negative breast cancer were treated as separate diseases for these analyses.

Sensitivity analysis showed that excluding the ASPREE controls gave broadly similar adjusted OR estimates as the main analyses, though with wider confidence intervals (Supplementary Table 7), validating our adjustment for age. Another sensitivity analysis showed that our results were almost unaffected by the exclusion of subjects with pathogenic variants in two or more genes (Supplementary Table 8).

## Discussion

The OR for breast cancer risk associated with a pathogenic variant in *BRCA1* and *BRCA2* in this study is consistent with other estimates^8^. These estimates are lower than reports that involve cases selected via criteria targeting breast and ovarian cancer syndromes and triple-negative breast cancer^12,13^. The breast cancer risk estimates reported here for *BRCA1* and *BRCA2* are less than the point estimates published by Dorling et al. and Hu et al. but are not statistically significantly different^9,10^.

Consistent with many other studies we found that, after pathogenic variants in *BRCA1* or *BRCA2*, pathogenic variants in *CHEK2* were the most frequently identified. The prevalence of *CHEK2* c.1100delC in some populations makes it possible for analyses to consider the risk associated with this variant individually. Although there is some evidence that this risk may be higher than that associated with all other *CHEK2* pathogenic variants, our estimates did not reach statistical significance. For instance, for breast cancer risk, Lu et al. estimated an OR of 4.00 [95% CI: 2.04–8.73] for *CHEK2* c.1100delC and an OR of 1.42 [95% CI: 0.76–2.81] for all other *CHEK2* pathogenic variants in their study of 11,416 affected women^16^ and Dorling et al. estimated an OR of 2.66 [95% CI: 2.27–3.11] for *CHEK2* c.1100delC and an OR of 2.13 [95% CI: 1.60–2.84] for all other *CHEK2* pathogenic variants in the population-based setting of their study^10^. Our study also found no statistical difference between c.1100delC (OR 1.1 [95% CI: 0.29–3.8]) and all other CHEK2 pathogenic variants (*p* = 0.75). *CHEK2* pathogenic variant carriers are an important group of women to identify as they are at an increased risk of contralateral breast cancer and have a lower survival compared with non-*CHEK2* pathogenic variant carriers (these data are predominantly informed by information about *CHEK2* c.1100delC^17–22^) and could benefit from specific screening modalities such as magnetic resonance imaging^23^.

We identified 17 carriers of pathogenic variants in *ATM*, only one of which was *ATM* c.7271T > G, a pathogenic variant that is well described in the Australian population and has an established association with a substantially increased risk of breast cancer^24–26^. Breast cancer risk estimates for women carrying other pathogenic variants in *ATM* have been consistently reported to be in the order of 2–3-fold^8–10,12–14,16^.

The literature has consistently reported a prevalence of germline pathogenic variants in genes other than *BRCA1* and *BRCA2* of ~4% when affected women attending high-risk clinics are the study subjects and gene panel tests are applied^1,2^. The prevalence of pathogenic variants in genes other than *BRCA1* or *BRCA2* are also similar in reports from clinical series of affected women (unselected for family history)^4^. Our findings are consistent with previous work in this setting and illustrate that, in contrast to pathogenic variants in *BRCA1* and *BRCA2* which are less prevalent in women diagnosed at older ages, the prevalence of pathogenic variants in other breast cancer genes is independent of age at diagnosis^4,9^.

In our Australian population-based case subjects, the frequency of pathogenic variants in genes other than *BRCA1* or *BRCA2* was 5%, as high as that reported from groups of women from high-risk clinical settings. This frequency may be surprising given the population-based ABCFS recruited participants unselected for family history. However, other attributes of the ABCFS participants and the nature of the breast cancer risks associated with pathogenic variants in genes other than *BRCA1* and *BRCA2* may partially provide an explanation. Predictive factors used to identify families appropriate to refer to high-risk genetics clinics, which include family history, have not been found to be as predictive for carrying pathogenic variants in genes other than *BRCA1* and *BRCA2*^4^. In addition, the average penetrance of pathogenic variants in these genes, although not precisely estimated beyond *PALB2*^27,28^, is anticipated to be lower than for pathogenic variants in *BRCA1* and *BRCA2*. The relative risks estimated here and elsewhere support this expectation. Therefore, by not selecting for affected women with a family history, yet having a focus on early onset disease, the ABCFS has a prevalence of pathogenic variants in other breast cancer susceptibility genes similar to that of highly selected women attending genetics services. Improved predictors for identifying women and families who carry pathogenic variants in breast cancer predisposition genes other than *BRCA1* and *BRCA2* are urgently needed.

A significant challenge for studies in this setting, and the assessment of very rare variants, is the availability of suitable datasets to use as reference controls. Few have had resources that provide population-specific reference control datasets of suitable size to incorporate into risk estimation methodology^29,30^. Large publicly available databases have recently become available, including ExAC and gnomAD. Although they constitute invaluable resources as variant frequency databases and can be used to filter out “common” variants that are unlikely to be associated with increased risk of disease, these databases have important limitations when used as controls in case-control studies^7,12^. These limitations include (i) potential technical artefacts resulting from the aggregation of data generated by different sequencing platforms, (ii) differences in the call sets due to the cases and controls not being jointly processed or annotated, (iii) the absence or limited lifestyle and ancestry information for the control subjects and (iv) the absence of genetic information available at the individual subject level as only variant-based data is available. In the context of gene-burden analysis for rare conditions, these public databases can serve as reasonable control datasets with additional computational precautions to mitigate the above-mentioned issues, as described by Guo et al.^31^. For common diseases including cancer, they are still likely to contain affected individuals, even when excluding the TCGA sample set of gnomAD and ExAC. By using 862 population-based age-match controls from the ABCFS and 6549 older healthy Australian women participating in ASPREE, our study overcame some of these limitations.

The inclusion of older controls from ASPREE in this study means that our unadjusted OR estimates are biased, so we adjusted all ORs in our main analyses for age and other potential confounders (though we note that our unadjusted p-values are valid, since ascertainment bias disappears under the null hypothesis in our case, and super-cases and super-controls can validly be used for gene discovery). A sensitivity analysis based just on the age-matched cases and controls from the ABCFS gave broadly similar ORs as the main analyses, validating our adjustment methods and our adjusted OR estimates.

In our study, although different sequencing platforms have been used to generate the raw sequencing data, we aimed to reduce potential artefactual variant calls by utilising the processing pipeline that was the most appropriate for the sequencing technology used to produce the raw sequencing data for the case and the control subjects, then harmonising the variant calls by (i) restricting calls to regions that are equally able to be called across the three targeted regions and (ii) applying the same filtering and annotation pipelines. Our study used ClinVar to select pathogenic variants to include, as a group, in our association analysis. Although the level of confidence in ClinVar calls can be variable, as demonstrated by the star rating system or the “Conflicting evidence of pathogenicity” label, this approach allowed us to harmonise our pathogenicity calls with other studies, e.g., from Ambry^8^ or Myriad^11^ who regularly deposit their classification calls into ClinVar. For genes such as *BRCA1*, *BRCA2*, *TP53* or the mismatch repair genes, we were also able to keep our pathogenicity assessment contemporary with regular updates from the genes respective expert panels.

A limitation of our approach is the potential to underestimate the contribution of missense variants, as they are very challenging to classify. Functional assays can provide important additional information for variant classification but are currently less well developed for breast cancer predisposition genes other than *BRCA1* and *BRCA2*, although some recent and promising progress has been made for *PALB2*^32^. A large number of unclassified variants (*n* = 924) were identified in the case subjects of the ABCFS in this study. It is likely that an extremely small number of these variants will be classified as pathogenic in the future. Recent data from Dorling et al.^10^, provided further evidence for breast cancer risk for missense variants in a number of breast cancer susceptibility genes, most notably *CHEK2*^10,33^. Considerable effort has been invested by the ENIGMA consortium to understand the effect of deleterious variants in these genes and keep the variant classification up-to-date and publicly available.

Our data provide a population perspective to gene panel testing for breast cancer predisposition and contribute to international efforts to refine the breast cancer risk estimates for genetic variants identified in panel testing in women enriched for early age at breast cancer diagnosis and unselected for family history.

## Methods

### Subjects

The present study includes cases and controls from the Australian Breast Cancer Family Study (ABCFS) and participants from the ASPirin in Reducing Events in the Elderly (ASPREE) study.

Aspects of the ABCFS relevant to this study are the population-based probands and corresponding data collected at baseline. Briefly, the ABCFS probands were either breast cancer cases (identified through population-complete cancer registries) or age-matched controls. All probands completed interviewer-administered risk factor questionnaires and verification of cancers was sought through pathologist reviews of cancer tissue, pathology reports, cancer registries, medical records, and death certificates^34,35^.

The ASPREE study is a randomized, placebo-controlled trial for daily low-dose aspirin. We selected Australian participants aged 70 years or older at enrolment, without a previous diagnosis or current symptoms of atherothrombotic cardiovascular disease, physical disability, or dementia. Study design, recruitment, baseline characteristics and outcomes have been previously described^36,37^. Our statistical analysis only used ASPREE data that were collected at baseline. ASPREE female participants who reported at baseline a personal history of breast cancer were excluded from the statistical analysis.

Written informed consent was obtained from all individual participants included in the study. This study was approved by the Human Research Ethics Committee of the University of Melbourne and Monash University.

### Gene panel testing

We analysed rare genetic variants identified in the blood-derived germline DNA of 1,451 women diagnosed with breast cancer and 857 age-matched controls participating in the ABCFS, and 13,197 individuals (6549 women) enroled in the ASPREE trial.

### Genes included in the gene panel

Our analysis was restricted to the coding region and proximal intron-exon junctions of 24 genes; *ATM*: NM_000051, *BARD1*: NM_000465.2, *BRCA1*: NM_007294.3, *BRCA2*: NM_000059.3, *BRIP1*: NM_032043.2, *CDH1*: NM_004360.3, *CHEK2*: NM_007194.3, *FANCM*: NM_020937.2, *MLH1*: NM_000249.3, *MRE11A*: NM_005591.3, *MSH2*: NM_000251.2, *MSH6*: NM_000179.2, *MUTYH*: NM_001128425.1, *NBN*: NM_002485.4, *NF1*: NM_000267.3, *PALB2*: NM_024675.3, *PMS2*: NM_000535.5, *PTEN*: NM_000314.4, *RAD50*: NM_005732.3, *RAD51C*: NM_058216.2, *RAD51D*: NM_002878.3, *RECQL*: NM_002907.3, *STK11*: NM_000455.4, *TP53*: NM_000546.5.

Only selected regions of PMS2 were targeted as described previously^38^. Gene-panel testing and raw DNA sequencing reads alignment to the reference genome GRCh37 were performed as described in Nguyen-Dumont et al. and Lacaze et al. for the ABCFS and ASPREE subjects, respectively^38,39^. Briefly, the ABCFS subjects were sequenced in-house, using either a Hi-Plex panel on the NextSeq550^40^ or a HaloPlexHS panel on the HiSeq3000 (both Illumina, San Diego, CA, USA). The ASPREE subjects were sequenced using an AmpliSeq panel on the Ion Torrent S5TM XL (Thermo Fisher Scientific, Waltham, MA, USA) and aligned sequencing files (BAMs) were provided for variant calling in this study.

### Variant calling and filtering

Variant calling was performed using VarDict 1.7^41^ and restricted to the overlap of the regions targeted by the three panels. For ASPREE controls sequenced on the Ion Torrent platform, variant calling had also been performed using the Torrent Variant Calling Suite v1.5 as previously described^42^ and the intersection with the variant calls from VarDict was used in downstream analyses. Subsequent genetic analyses were restricted to variants: (i) with the following read depth and variant allele frequency: 50X and 0.2 for Hi-Plex and AmpliSeq samples, and 30X and 0.15 for HaloPlexHS samples. In addition, for the ASPREE samples, we determined a conservative but high-confidence call set by filtering out (i) variants present in more than 0.05% of all ASPREE participants (*n* = 65), under the assumption that common variants were either sequencing artefacts or too common to be associated with disease risk, and (ii) variants that had passed our quality filters described above in <95% of the genotype calls at a given genomic location, to ensure that variants that progressed to the next analysis stage were adequately covered.

Variant annotation was performed using VarSeq VSClinical v2.2 (Golden Helix Inc., Bozeman, MT, USA) and included ClinVar annotations from July 2020. This study focused on rare predicted protein-truncating variants (PTVs) and pathogenic (including likely pathogenic) variants. Rare variants were defined as those identified in ExAC v.0.3 with a minor allele frequency ≤0.01 in the non-Finnish European population (NFE non-TCGA). Genetic variants were considered pathogenic if they were annotated as “Pathogenic” or “Likely Pathogenic” in ClinVar. Mono-allelic pathogenic *MUTYH* variant carriers are reported in Supplementary Table 1 but not included in our analysis. Predicted PTVs that were classified as “Conflicting” in ClinVar with annotations tending towards pathogenicity (e.g., *CHEK2* c.1100delC) were included in this analysis. Also included were PTVs that were absent (unreported) in ClinVar, except if they were located in the last coding exon. Further details can be found in Supplementary Table 1.

### Statistical methods

For each of the genes considered, pathogenic variants were combined and an odds ratio (OR) for their association with breast cancer was estimated using unconditional multivariate logistic regression. These analyses were adjusted for the following potential confounders (or, where indicated, a subset of these): age at enrolment, height, body mass index, number of children, number of years of education and number of alcoholic drinks per week. These potential confounders are the known breast cancer risk factors that are available in both the ABCFS and ASPREE datasets, and data harmonisation was performed to make the relevant variables from these two studies comparable.

Excluded from all statistical analyses were males, ASPREE females with a previous diagnosis of breast cancer, and females with no gene-panel testing data. Women with missing data were excluded from analyses involving the relevant variables, though <1% had missing values for each variable (except for number of children, which was missing for 4.3% of ASPREE). Women with pathogenic variants in two or more genes (other than *MUTYH*, see above) were excluded from the main analyses.

The effect of exclusions and other analytical choices was investigated with sensitivity analyses. Wald confidence intervals were calculated for each OR, and the likelihood ratio test was used to generate *p*-values for comparing nested models. All *p*-values were two-sided and a *p*-value threshold of 0.05 was used to define statistical significance. All analyses were performed using R version 3.4.2.

### Reporting summary

Further information on research design is available in the Nature Research Reporting Summary linked to this article.

## Supplementary information

Supplementary Information

Reporting Summary

## Data Availability

All rare pathogenic variants identified and the corresponding phenotype data are reported in the Supplementary Tables.
