# Supplementary material for: Population-based estimates of breast cancer risk for carriers of pathogenic variants identified by gene-panel testing
Source: NPJ Breast Cancer. 2021 Dec 9;7:153. doi: 10.1038/s41523-021-00360-3 (PMC8660783; doi:10.1038/s41523-021-00360-3)
Supplement: Supplementary file 1 — Supplementary Information [file 41523_2021_360_MOESM1_ESM.pdf]

Supplementary Table 1

Pathogenic[1] variants carriers identified by gene-panel testing in affected cases participating in the Australian Breast Cancer Family Study (ABCFS).

Gene	impact	HGVSc	HGVSp	Number of carriers
**ATM**	frameshift_variant	NM_000051.3:c.1355del	NP_000042.3:p.Thr452Asnfs*21	1
**ATM**	stop_gained	NM_000051.3:c.1396C>T	NP_000042.3:p.Gln466Ter	1
**ATM**	frameshift_variant	NM_000051.3:c.3802del	NP_000042.3:p.Val1268Terfs	1
**ATM**	frameshift_variant	NM_000051.3:c.5156del	NP_000042.3:p.Asn1719Ilefs*5	1
**ATM**	stop_gained	NM_000051.3:c.5515C>T	NP_000042.3:p.Gln1839Ter	1
**ATM**	frameshift_variant	NM_000051.3:c.5712dup	NP_000042.3:p.Ser1905Ilefs*25	1
**ATM**	frameshift_variant	NM_000051.3:c.6671dup	NP_000042.3:p.Met2224Ilefs*25,.	1
**ATM**	missense_variant	NM_000051.3:c.7271T>G	NP_000042.3:p.Val2424Gly,.	1
**ATM**	stop_gained	NM_000051.3:c.7792C>T	NP_000042.3:p.Arg2598Ter,.	1
**ATM**	frameshift_variant	NM_000051.3:c.7957_7960dup	NP_000042.3:p.Thr2654Asnfs*3,.	1
**ATM**	stop_gained	NM_000051.3:c.8098A>T	NP_000042.3:p.Lys2700Ter,.	1
**ATM**	missense_variant	NM_000051.3:c.8122G>A	NP_000042.3:p.Asp2708Asn,.	1
**ATM**	splice_donor_variant	NM_000051.3:c.8418+5_8418+8del	.,.	2
**ATM**	missense_variant	NM_000051.3:c.8494C>T	NP_000042.3:p.Arg2832Cys,.	1
**ATM**	stop_gained	NM_000051.3:c.9139C>T	NP_000042.3:p.Arg3047Ter,.	1
**BARD1**	stop_gained	NM_000465.2:c.1652C>G	NP_000456.2:p.Ser551Ter	1
**BARD1**	stop_gained	NM_000465.2:c.298C>T	NP_000456.2:p.Gln100Ter	1
**BARD1**	frameshift_variant	NM_000465.2:c.627_628del	NP_000456.2:p.Lys209Asnfs*4	1
**BRCA1**	frameshift_variant	NM_007294.3:c.1287dup	NP_009225.1:p.Asp430Argfs*6	1
**BRCA1**	missense_variant	NM_007294.3:c.131G>T	NP_009225.1:p.Cys44Phe	1
**BRCA1**	splice_acceptor_variant	NM_007294.3:c.135-1G>T	.	1
**BRCA1**	stop_gained	NM_007294.3:c.1687C>T	NP_009225.1:p.Gln563Ter	1
**BRCA1**	frameshift_variant	NM_007294.3:c.1757del	NP_009225.1:p.Pro586Leufs*2	1

**BRCA1**	stop_gained	NM_007294.3:c.1840A>T	NP_009225.1:p.Lys614Ter	1
**BRCA1**	splice_donor_variant	NM_007294.3:c.212+1G>T	.	1
**BRCA1**	frameshift_variant	NM_007294.3:c.2389_2390del	NP_009225.1:p.Glu797Thrfs*3	1
**BRCA1**	frameshift_variant	NM_007294.3:c.2475del	NP_009225.1:p.Asp825Glufs*21	2
**BRCA1**	frameshift_variant	NM_007294.3:c.2681_2682del	NP_009225.1:p.Lys894Thrfs*8	2
**BRCA1**	stop_gained	NM_007294.3:c.2800C>T	NP_009225.1:p.Gln934Ter	1
**BRCA1**	splice_acceptor_variant	NM_007294.3:c.302-2del	.	2
**BRCA1**	stop_gained	NM_007294.3:c.303T>G	NP_009225.1:p.Tyr101Ter	1
**BRCA1**	frameshift_variant	NM_007294.3:c.3155del	NP_009225.1:p.Asn1052Metfs*10	1
**BRCA1**	frameshift_variant	NM_007294.3:c.3296del	NP_009225.1:p.Pro1099Leufs*10	1
**BRCA1**	frameshift_variant	NM_007294.3:c.3331_3334del	NP_009225.1:p.Gln1111Asnfs*5	1
**BRCA1**	frameshift_variant	NM_007294.3:c.3700_3704del	NP_009225.1:p.Val1234Glnfs*8	1
**BRCA1**	frameshift_variant	NM_007294.3:c.3756_3759del	NP_009225.1:p.Ser1253Argfs*10	1
**BRCA1**	frameshift_variant	NM_007294.3:c.3770_3771del	NP_009225.1:p.Glu1257Glyfs*9	1
**BRCA1**	frameshift_variant	NM_007294.3:c.4065_4068del	NP_009225.1:p.Asn1355Lysfs*10	1
**BRCA1**	frameshift_variant	NM_007294.3:c.4239del	NP_009225.1:p.Glu1413Aspfs*2	1
**BRCA1**	stop_gained	NM_007294.3:c.427G>T	NP_009225.1:p.Glu143Ter	2
**BRCA1**	stop_gained	NM_007294.3:c.4327C>T	NP_009225.1:p.Arg1443Ter	2
**BRCA1**	splice_donor_variant	NM_007294.3:c.4675+1G>A	.	1
**BRCA1**	stop_gained	NM_007294.3:c.4689C>G	NP_009225.1:p.Tyr1563Ter	2
**BRCA1**	missense_variant	NM_007294.3:c.5057A>G	NP_009225.1:p.His1686Arg	1
**BRCA1**	missense_variant	NM_007294.3:c.5095C>T	NP_009225.1:p.Arg1699Trp	1
**BRCA1**	frameshift_variant	NM_007294.3:c.5177_5180del	NP_009225.1:p.Arg1726Lysfs*3	1
**BRCA1**	frameshift_variant	NM_007294.3:c.5266dup	NP_009225.1:p.Gln1756Profs*74	6
**BRCA1**	frameshift_variant	NM_007294.3:c.68_69del	NP_009225.1:p.Glu23Valfs*17	1
**BRCA1**	frameshift_variant	NM_007294.3:c.70_80del	NP_009225.1:p.Cys24Serfs*13	2
**BRCA1**	splice_donor_variant	NM_007294.3:c.80+2T>G	.	1
**BRCA2**	stop_gained	NM_000059.3:c.250C>T	NP_000050.2:p.Gln84Ter	2
**BRCA2**	frameshift_variant	NM_000059.3:c.2808_2811del	NP_000050.2:p.Ala938Profs*21	2
**BRCA2**	frameshift_variant	NM_000059.3:c.3519_3523del	NP_000050.2:p.Gln1175Argfs*7	1

**BRCA2**	frameshift_variant	NM_000059.3:c.3847_3848del	NP_000050.2:p.Val1283Lysfs*2	2
**BRCA2**	frameshift_variant	NM_000059.3:c.4163_4164delinsA	NP_000050.2:p.Thr1388Asnfs*22	1
**BRCA2**	frameshift_variant	NM_000059.3:c.4631del	NP_000050.2:p.Asn1544Thrfs*24	2
**BRCA2**	stop_gained	NM_000059.3:c.4859T>G	NP_000050.2:p.Leu1620Ter	1
**BRCA2**	frameshift_variant	NM_000059.3:c.5410_5411del	NP_000050.2:p.Val1804Lysfs*2	1
**BRCA2**	frameshift_variant	NM_000059.3:c.5576_5579del	NP_000050.2:p.Ile1859Lysfs*3	1
**BRCA2**	frameshift_variant	NM_000059.3:c.5946del	NP_000050.2:p.Ser1982Argfs*22	6
**BRCA2**	stop_gained	NM_000059.3:c.6065C>G	NP_000050.2:p.Ser2022Ter	1
**BRCA2**	frameshift_variant	NM_000059.3:c.6275_6276del	NP_000050.2:p.Leu2092Profs*7	4
**BRCA2**	frameshift_variant	NM_000059.3:c.7177dup	NP_000050.2:p.Met2393Asnfs*19	1
**BRCA2**	frameshift_variant	NM_000059.3:c.755_758del	NP_000050.2:p.Asp252Valfs*24	2
**BRCA2**	splice_acceptor_variant	NM_000059.3:c.7806-2A>G	.	1
**BRCA2**	splice_acceptor_variant	NM_000059.3:c.7977-1G>C	.	1
**BRCA2**	missense_variant	NM_000059.3:c.8167G>C	NP_000050.2:p.Asp2723His	2
**BRCA2**	frameshift_variant	NM_000059.3:c.8575del	NP_000050.2:p.Gln2859Lysfs*4	2
**BRCA2**	stop_gained	NM_000059.3:c.8695C>T	NP_000050.2:p.Gln2899Ter	1
**BRCA2**	stop_gained	NM_000059.3:c.8869C>T	NP_000050.2:p.Gln2957Ter	1
**BRCA2**	stop_gained	NM_000059.3:c.8878C>T	NP_000050.2:p.Gln2960Ter	1
**BRCA2**	frameshift_variant	NM_000059.3:c.8904del	NP_000050.2:p.Val2969Cysfs*7	1
**BRCA2**	splice_region_variant	NM_000059.3:c.9117G>A	NP_000050.2:p.Pro3039	1
**BRCA2**	stop_gained	NM_000059.3:c.9276T>G	NP_000050.2:p.Tyr3092Ter	1
**BRCA2**	stop_gained	NM_000059.3:c.9294C>G	NP_000050.2:p.Tyr3098Ter	1
**BRIP1**	stop_gained	NM_032043.2:c.1058dup	NP_114432.2:p.Tyr353Terfs	1
**BRIP1**	stop_gained	NM_032043.2:c.1343G>A	NP_114432.2:p.Trp448Ter	1
**BRIP1**	frameshift_variant	NM_032043.2:c.1956del	NP_114432.2:p.Ser653Glnfs*35	1
**BRIP1**	frameshift_variant	NM_032043.2:c.2255_2256del	NP_114432.2:p.Lys752Argfs*12	1
**BRIP1**	stop_gained	NM_032043.2:c.2392C>T	NP_114432.2:p.Arg798Ter	2
**BRIP1**	frameshift_variant	NM_032043.2:c.2992_2995del	NP_114432.2:p.Lys998Glufs*60	1
**BRIP1**	frameshift_variant	NM_032043.2:c.394dup	NP_114432.2:p.Thr132Asnfs*10	1
**CHEK2**	frameshift_variant	NM_007194.3:c.1100del	NP_009125.1:p.Thr367Metfs*15	9

**CHEK2**	frameshift_variant	NM_007194.3:c.1263del	NP_009125.1:p.Ser422Valfs*15	2
**CHEK2**	stop_gained	NM_007194.3:c.1528C>T	NP_009125.1:p.Gln510Ter	1
**CHEK2**	splice_donor_variant	NM_007194.3:c.319+2T>A	.	1
**CHEK2**	missense_variant	NM_007194.3:c.349A>G	NP_009125.1:p.Arg117Gly	3
**CHEK2**	frameshift_variant	NM_007194.3:c.405del	NP_009125.1:p.Lys135Asnfs*26	1
**CHEK2**	splice_donor_variant	NM_007194.3:c.444+1G>A	.	1
**CHEK2**	stop_gained	NM_007194.3:c.823G>T	NP_009125.1:p.Glu275Ter	1
**CHEK2**	frameshift_variant	NM_007194.3:c.920dup	NP_009125.1:p.Glu308Argfs*4	1
**FANCM**	frameshift_variant	NM_020937.2:c.3843dup	NP_065988.1:p.Pro1282Thrfs*5	2
**FANCM**	splice_donor_variant	NM_020937.2:c.4317+1G>T	.	1
**FANCM**	stop_gained	NM_020937.2:c.5791C>T	NP_065988.1:p.Arg1931Ter	1
**MSH6**	stop_gained	NM_000179.2:c.272C>A	NP_000170.1:p.Ser91Ter	1
**MSH6**	frameshift_variant	NM_000179.2:c.3261dup	NP_000170.1:p.Phe1088Leufs*5	1
**MSH6**	stop_gained	NM_000179.2:c.3526A>T	NP_000170.1:p.Arg1176Ter	1
**MUTYH**	missense_variant	NM_001128425.1:c.1187G>A	NP_001121897.1:p.Gly396Asp	8
**MUTYH**	stop_gained	NM_001128425.1:c.312C>A	NP_001121897.1:p.Tyr104Ter	1
**MUTYH**	missense_variant	NM_001128425.1:c.536A>G	NP_001121897.1:p.Tyr179Cys	7
**MUTYH**	stop_gained	NM_001128425.1:c.55C>T	NP_001121897.1:p.Arg19Ter	1
**NBN**	stop_gained	NM_002485.4:c.143T>G	NP_002476.2:p.Leu48Ter	1
**NF1**	frameshift_variant	NM_000267.3:c.1837_1841del	NP_000258.1:p.Lys613Terfs	1
**NF1**	frameshift_variant	NM_000267.3:c.3525_3526del	NP_000258.1:p.Arg1176Serfs*18	1
**PALB2**	frameshift_variant	NM_024675.3:c.1050_1053del	NP_078951.2:p.Thr351Argfs*4	1
**PALB2**	frameshift_variant	NM_024675.3:c.2052del	NP_078951.2:p.Arg686Glyfs*23	1
**PALB2**	frameshift_variant	NM_024675.3:c.2167_2168del	NP_078951.2:p.Met723Valfs*21	1
**PALB2**	stop_gained	NM_024675.3:c.2718G>A	NP_078951.2:p.Trp906Ter	1
**PALB2**	stop_gained	NM_024675.3:c.3113G>A	NP_078951.2:p.Trp1038Ter	2
**PALB2**	stop_gained	NM_024675.3:c.3256C>T	NP_078951.2:p.Arg1086Ter	1
**RAD50**	stop_gained	NM_005732.3:c.1875C>G	NP_005723.2:p.Tyr625Ter	1
**RAD51D**	frameshift_variant	NM_002878.3:c.748del	NP_002869.3:p.His250Thrfs*2	1
**RAD51D**	frameshift_variant	NM_002878.3:c.85del	NP_002869.3:p.Val29Trpfs*11	1

**RECQL**	splice_acceptor_variant	NM_002907.3:c.868-2A>G	.	1
**TP53**	splice_region_variant	NM_000546.5:c.375G>A	NP_000537.3:p.Thr125	1
**TP53**	missense_variant	NM_000546.5:c.524G>A	NP_000537.3:p.Arg175His	1
**TP53**	splice_donor_variant	NM_000546.5:c.559+2T>G	.	1
**TP53**	missense_variant	NM_000546.5:c.641A>G	NP_000537.3:p.His214Arg	1
**TP53**	frameshift_variant	NM_000546.5:c.731del	NP_000537.3:p.Gly244Alafs*3	1
**TP53**	missense_variant	NM_000546.5:c.844C>T	NP_000537.3:p.Arg282Trp	1

[1] Pathogenic (including likely pathogenic) as defined by ClinVar and protein truncating variants that are absent from ClinVar (accessed July 2020). Excludes carriers of a protein truncating variants located in the last coding exon.

Supplementary Table 2

Pathogenic[1] variants carriers identified by gene-panel testing in unaffected controls participating in the Australian Breast Cancer Family Study (ABCFS).

gene	impact	HGVSc	HGVSp	Number of carriers
**ATM**	splice_acceptor_variant	NM_000051.3:c.3078-1G>A	.	1
**ATM**	inframe_deletion	NM_000051.3:c.7638_7646del	NP_000042.3:p.Arg2547_Ser2549del,.	1
**ATM**	frameshift_variant	NM_000051.3:c.8264_8268del	NP_000042.3:p.Tyr2755Cysfs*12,.	1
**BRCA2**	splice_donor_variant	NM_000059.3:c.516+1G>T	.	1
**BRCA2**	frameshift_variant	NM_000059.3:c.6275_6276del	NP_000050.2:p.Leu2092Profs*7	1
**BRCA2**	frameshift_variant	NM_000059.3:c.6405_6409del	NP_000050.2:p.Asn2135Lysfs*3	1
**BRCA2**	stop_gained	NM_000059.3:c.6952C>T	NP_000050.2:p.Arg2318Ter	2
**BRCA2**	frameshift_variant	NM_000059.3:c.8575del	NP_000050.2:p.Gln2859Lysfs*4	1
**MLH1**	splice_acceptor_variant	NM_000249.3:c.1668-1G>A	.	1
**MSH2**	stop_gained	NM_000251.2:c.892C>T	NP_000242.1:p.Gln298Ter	1
**MUTYH**	frameshift_variant	NM_001128425.1:c.1147del	NP_001121897.1:p.Ala385Profs*23	1
**MUTYH**	missense_variant	NM_001128425.1:c.1187G>A	NP_001121897.1:p.Gly396Asp	4
**MUTYH**	missense_variant	NM_001128425.1:c.325C>T	NP_001121897.1:p.Arg109Trp	1
**MUTYH**	missense_variant	NM_001128425.1:c.536A>G	NP_001121897.1:p.Tyr179Cys	1
**MUTYH**	splice_region_variant	NM_001128425.1:c.690G>A	NP_001121897.1:p.Gln230	1
**RAD51D**	stop_gained	NM_002878.3:c.803G>A	NP_002869.3:p.Trp268Ter	1
**RECQL**	stop_gained	NM_002907.3:c.730C>T	NP_002898.2:p.Gln244Ter	1
**RAD50**	stop_gained	NM_005732.3:c.1393C>T	NP_005723.2:p.Gln465Ter	2
**RAD50**	frameshift_variant	NM_005732.3:c.2938_2942del	NP_005723.2:p.Leu980Terfs	1
**RAD50**	frameshift_variant	NM_005732.3:c.3440_3441dup	NP_005723.2:p.Arg1148Tyrfs*17	1
**CHEK2**	frameshift_variant	NM_007194.3:c.1100del	NP_009125.1:p.Thr367Metfs*15	3
**CHEK2**	missense_variant	NM_007194.3:c.349A>G	NP_009125.1:p.Arg117Gly	3

**CHEK2**	frameshift_variant	NM_007194.3:c.591del	NP_009125.1:p.Val198Phefs*7	1
**BRCA1**	missense_variant	NM_007294.3:c.140G>A	NP_009225.1:p.Cys47Tyr	1
**BRCA1**	splice_acceptor_variant	NM_007294.3:c.302-2del	.	1
**BRCA1**	missense_variant	NM_007294.3:c.5363G>T	NP_009225.1:p.Gly1788Val	1
**BRCA1**	frameshift_variant	NM_007294.3:c.68_69del	NP_009225.1:p.Glu23Valfs*17	1
**FANCM**	stop_gained	NM_020937.2:c.5791C>T	NP_065988.1:p.Arg1931Ter	1
**FANCM**	splice_donor_variant	NM_020937.2:c.6008+1G>A	.	1
**PALB2**	frameshift_variant	NM_024675.3:c.3116del	NP_078951.2:p.Asn1039Ilefs*2	1
**BRIP1**	frameshift_variant	NM_032043.2:c.394dup	NP_114432.2:p.Thr132Asnfs*10	1
**BRIP1**	frameshift_variant	NM_032043.2:c.608del	NP_114432.2:p.Asn203Thrfs*71	1
**RAD51C**	missense_variant	NM_058216.2:c.773G>A	NP_478123.1:p.Arg258His	1

[1] Pathogenic (including likely pathogenic) as defined by ClinVar and protein truncating variants that are absent from ClinVar (accessed July 2020). Excludes carriers of a protein truncating variants located in the last coding exon.

Supplementary Table 3

Pathogenic[1] variants carriers identified by gene-panel testing in women participating in the ASPirin in Reducing Events in the Elderly (ASPREE) study.

gene	impact	HGVSc	HGVSp	Number of carriers
**ATM**	frameshift_variant	NM_000051.3:c.1564_1565del	NP_000042.3:p.Glu522Ilefs*43	1
**ATM**	frameshift_variant	NM_000051.3:c.15dup	NP_000042.3:p.Asn6Terfs	1
**ATM**	frameshift_variant	NM_000051.3:c.1685del	NP_000042.3:p.Asn562Ilefs*5	3
**ATM**	stop_gained	NM_000051.3:c.2098C>T	NP_000042.3:p.Gln700Ter	1
**ATM**	splice_region_variant	NM_000051.3:c.2250G>A	NP_000042.3:p.Lys750	1
**ATM**	frameshift_variant	NM_000051.3:c.2284_2285del	NP_000042.3:p.Leu762Valfs*2	1
**ATM**	stop_gained	NM_000051.3:c.2426C>A	NP_000042.3:p.Ser809Ter	2
**ATM**	frameshift_variant	NM_000051.3:c.2483del	NP_000042.3:p.Lys828Serfs*8	1
**ATM**	splice_acceptor_variant	NM_000051.3:c.3078-1G>A	.	1
**ATM**	frameshift_variant	NM_000051.3:c.3130_3131dup	NP_000042.3:p.Asn1044Lysfs*21	1
**ATM**	frameshift_variant	NM_000051.3:c.3131dup	NP_000042.3:p.Asn1044Lysfs*4	1
**ATM**	stop_gained	NM_000051.3:c.3756T>A	NP_000042.3:p.Tyr1252Ter	1
**ATM**	frameshift_variant	NM_000051.3:c.3802del	NP_000042.3:p.Val1268Terfs	2
**ATM**	frameshift_variant	NM_000051.3:c.3850del	NP_000042.3:p.Thr1284Glnfs*9	1
**ATM**	splice_acceptor_variant	NM_000051.3:c.4111dup	NP_000042.3:p.Asp1371Glyfs*7	1
**ATM**	stop_gained	NM_000051.3:c.5623C>T	NP_000042.3:p.Arg1875Ter	2
**ATM**	stop_gained	NM_000051.3:c.5771C>A	NP_000042.3:p.Ser1924Ter	1
**ATM**	splice_donor_variant	NM_000051.3:c.5918+1G>A	.	1
**ATM**	frameshift_variant	NM_000051.3:c.6019_6020del	NP_000042.3:p.Glu2007Asnfs*10	1
**ATM**	frameshift_variant	NM_000051.3:c.6916_6917del	NP_000042.3:p.Leu2307Cysfs*65	2
**ATM**	frameshift_variant	NM_000051.3:c.6997dup	NP_000042.3:p.Thr2333Asnfs*40	1
**ATM**	missense_variant	NM_000051.3:c.7271T>G	NP_000042.3:p.Val2424Gly	2

**ATM**	frameshift_variant	NM_000051.3:c.7389dup	NP_000042.3:p.Cys2464Metfs*2	1
**ATM**	inframe_deletion	NM_000051.3:c.7638_7646del	NP_000042.3:p.Arg2547_Ser2549del	3
**ATM**	frameshift_variant	NM_000051.3:c.7829_7830del	NP_000042.3:p.Arg2610Lysfs*2	1
**ATM**	frameshift_variant	NM_000051.3:c.7886_7890del	NP_000042.3:p.Ile2629Serfs*25	1
**ATM**	stop_gained	NM_000051.3:c.7921C>T	NP_000042.3:p.Gln2641Ter	1
**ATM**	frameshift_variant	NM_000051.3:c.8101del	NP_000042.3:p.Ile2701Terfs	4
**ATM**	missense_variant	NM_000051.3:c.8122G>A	NP_000042.3:p.Asp2708Asn	1
**ATM**	frameshift_variant	NM_000051.3:c.8292_8293del	NP_000042.3:p.Ser2764Argfs*4	1
**ATM**	frameshift_variant	NM_000051.3:c.8305_8317del	NP_000042.3:p.Trp2769Leufs*33	1
**ATM**	stop_gained	NM_000051.3:c.8307G>A	NP_000042.3:p.Trp2769Ter	1
**ATM**	splice_donor_variant	NM_000051.3:c.8786+1G>A	.	2
**ATM**	frameshift_variant	NM_000051.3:c.8833_8834del	NP_000042.3:p.Leu2945Valfs*10	1
**ATM**	frameshift_variant	NM_000051.3:c.8873_8874del	NP_000042.3:p.Phe2958Terfs	1
**ATM**	stop_gained	NM_000051.3:c.9139C>T	NP_000042.3:p.Arg3047Ter	1
**ATM**	frameshift_variant	NM_000051.3:c.9146del	NP_000042.3:p.Phe3049Serfs	1
**BRCA2**	frameshift_variant	NM_000059.3:c.1189_1190insTTAG	NP_000050.2:p.Gln397Leufs*25	1
**BRCA2**	stop_gained	NM_000059.3:c.145G>T	NP_000050.2:p.Glu49Ter	1
**BRCA2**	frameshift_variant	NM_000059.3:c.1561del	NP_000050.2:p.Ser521Glnfs*4	7
**BRCA2**	frameshift_variant	NM_000059.3:c.2489dup	NP_000050.2:p.Asn830Lysfs*3	1
**BRCA2**	frameshift_variant	NM_000059.3:c.2808_2811del	NP_000050.2:p.Ala938Profs*21	1
**BRCA2**	stop_gained	NM_000059.3:c.314T>G	NP_000050.2:p.Leu105Ter	1
**BRCA2**	frameshift_variant	NM_000059.3:c.3170_3174del	NP_000050.2:p.Lys1057Thrfs*8	1
**BRCA2**	frameshift_variant	NM_000059.3:c.3680_3681del	NP_000050.2:p.Leu1227Glnfs*5	2
**BRCA2**	frameshift_variant	NM_000059.3:c.3847_3848del	NP_000050.2:p.Val1283Lysfs*2	1
**BRCA2**	frameshift_variant	NM_000059.3:c.3860dup	NP_000050.2:p.Asn1287Lysfs*2	1
**BRCA2**	frameshift_variant	NM_000059.3:c.4478_4481del	NP_000050.2:p.Glu1493Valfs*10	2
**BRCA2**	stop_gained	NM_000059.3:c.4648G>T	NP_000050.2:p.Glu1550Ter	1
**BRCA2**	frameshift_variant	NM_000059.3:c.495del	NP_000050.2:p.His166Ilefs*6	1
**BRCA2**	frameshift_variant	NM_000059.3:c.5130_5133del	NP_000050.2:p.Tyr1710Terfs	1
**BRCA2**	frameshift_variant	NM_000059.3:c.5297del	NP_000050.2:p.Asn1766Ilefs*11	3

**BRCA2**	frameshift_variant	NM_000059.3:c.5350_5351del	NP_000050.2:p.Asn1784Hisfs*2	1
**BRCA2**	frameshift_variant	NM_000059.3:c.5379_5385del	NP_000050.2:p.Asn1793Lysfs*10	1
**BRCA2**	frameshift_variant	NM_000059.3:c.5551dup	NP_000050.2:p.Ile1851Asnfs*7	1
**BRCA2**	stop_gained	NM_000059.3:c.5682C>G	NP_000050.2:p.Tyr1894Ter	1
**BRCA2**	stop_gained	NM_000059.3:c.5909C>A	NP_000050.2:p.Ser1970Ter	2
**BRCA2**	frameshift_variant	NM_000059.3:c.6405_6409del	NP_000050.2:p.Asn2135Lysfs*3	1
**BRCA2**	frameshift_variant	NM_000059.3:c.7069_7070del	NP_000050.2:p.Leu2357Valfs*2	2
**BRCA2**	missense_variant	NM_000059.3:c.7958T>C	NP_000050.2:p.Leu2653Pro	1
**BRCA2**	stop_gained	NM_000059.3:c.8174G>A	NP_000050.2:p.Trp2725Ter	1
**BRCA2**	frameshift_variant	NM_000059.3:c.8575del	NP_000050.2:p.Gln2859Lysfs*4	1
**BRCA2**	splice_acceptor_variant	NM_000059.3:c.8633-24_8634del	NP_000050.2:p.?	1
**BRCA2**	frameshift_variant	NM_000059.3:c.9157del	NP_000050.2:p.Glu3053Serfs*9	1
**BRCA2**	stop_gained	NM_000059.3:c.9294C>G	NP_000050.2:p.Tyr3098Ter	1
**BRCA2**	frameshift_variant	NM_000059.3:c.9699_9702del	NP_000050.2:p.Cys3233Trpfs*15	1
**MSH6**	frameshift_variant	NM_000179.2:c.1610_1613del	NP_000170.1:p.Lys537Ilefs*33	1
**MSH6**	frameshift_variant	NM_000179.2:c.1897del	NP_000170.1:p.Thr633Leufs*2	2
**MSH6**	frameshift_variant	NM_000179.2:c.2510del	NP_000170.1:p.His837Profs*7	1
**MSH6**	missense_variant	NM_000179.2:c.3188T>G	NP_000170.1:p.Leu1063Arg	1
**MSH6**	stop_gained	NM_000179.2:c.3202C>T	NP_000170.1:p.Arg1068Ter	1
**MSH6**	frameshift_variant	NM_000179.2:c.3238_3239del	NP_000170.1:p.Leu1080Valfs*12	1
**MSH6**	stop_gained	NM_000179.2:c.642C>G	NP_000170.1:p.Tyr214Ter	1
**MLH1**	missense_variant	NM_000249.3:c.350C>T	NP_000240.1:p.Thr117Met	1
**NF1**	splice_region_variant	NM_000267.3:c.1062+3A>G	.	1
**NF1**	frameshift_variant	NM_000267.3:c.233del	NP_000258.1:p.Asn78Ilefs*7	1
**NF1**	frameshift_variant	NM_000267.3:c.499_502del	NP_000258.1:p.Cys167Glnfs*10	1
**NF1**	frameshift_variant	NM_000267.3:c.603_604del	NP_000258.1:p.Phe201Leufs*14	1
**NF1**	frameshift_variant	NM_000267.3:c.7908_7909insT	NP_000258.1:p.His2637Serfs*2	2
**PTEN**	missense_variant	NM_000314.4:c.517C>T	NP_000305.3:p.Arg173Cys	1
**BARD1**	stop_gained	NM_000465.2:c.1212C>G	NP_000456.2:p.Tyr404Ter	1
**BARD1**	splice_acceptor_variant	NM_000465.2:c.159-1G>T	.	1

**BARD1**	stop_gained	NM_000465.2:c.1652C>G	NP_000456.2:p.Ser551Ter	5
**BARD1**	stop_gained	NM_000465.2:c.1905G>A	NP_000456.2:p.Trp635Ter	2
**BARD1**	frameshift_variant	NM_000465.2:c.614dup	NP_000456.2:p.Gln206Alafs*8	1
**BARD1**	frameshift_variant	NM_000465.2:c.627_628del	NP_000456.2:p.Lys209Asnfs*4	1
**TP53**	frameshift_variant	NM_000546.5:c.254del	NP_000537.3:p.Pro85Leufs*38	1
**TP53**	splice_acceptor_variant	NM_000546.5:c.376-2A>T	.	1
**NBN**	frameshift_variant	NM_002485.4:c.1142del	NP_002476.2:p.Pro381Glnfs*23	3
**NBN**	frameshift_variant	NM_002485.4:c.123del	NP_002476.2:p.Ser42Alafs*7	3
**NBN**	stop_gained	NM_002485.4:c.127C>T	NP_002476.2:p.Arg43Ter	1
**NBN**	frameshift_variant	NM_002485.4:c.156_157del	NP_002476.2:p.Ser53Cysfs*9	2
**NBN**	stop_gained	NM_002485.4:c.1741C>T	NP_002476.2:p.Gln581Ter	1
**NBN**	stop_gained	NM_002485.4:c.2140C>T	NP_002476.2:p.Arg714Ter	1
**NBN**	splice_donor_variant	NM_002485.4:c.2234+2T>G	.	1
**NBN**	frameshift_variant	NM_002485.4:c.657_661del	NP_002476.2:p.Lys219Asnfs*16	4
**NBN**	frameshift_variant	NM_002485.4:c.698_701del	NP_002476.2:p.Lys233Serfs*5	3
**NBN**	frameshift_variant	NM_002485.4:c.808_809del	NP_002476.2:p.Val270Cysfs*2	1
**RAD51D**	frameshift_variant	NM_002878.3:c.2dup	NP_002869.3:p.Met1Ilefs*70	1
**RAD51D**	frameshift_variant	NM_002878.3:c.564del	NP_002869.3:p.Val189Trpfs*5	1
**RAD51D**	stop_gained	NM_002878.3:c.571C>T	NP_002869.3:p.Gln191Ter	1
**RAD51D**	stop_gained	NM_002878.3:c.694C>T	NP_002869.3:p.Arg232Ter	1
**RAD51D**	splice_acceptor_variant	NM_002878.3:c.739-1G>C	.	1
**RAD51D**	frameshift_variant	NM_002878.3:c.748del	NP_002869.3:p.His250Thrfs*2	1
**RAD51D**	stop_gained	NM_002878.3:c.803G>A	NP_002869.3:p.Trp268Ter	1
**RAD51D**	stop_gained	NM_002878.3:c.898C>T	NP_002869.3:p.Arg300Ter	2
**CDH1**	splice_region_variant	NM_004360.3:c.1711+5G>A	.	1
**MRE11A**	stop_gained	NM_005591.3:c.1090C>T	NP_005582.1:p.Arg364Ter	2
**MRE11A**	frameshift_variant	NM_005591.3:c.1633_1640del	NP_005582.1:p.Leu545Tyrfs*9	1
**MRE11A**	stop_gained	NM_005591.3:c.1714C>T	NP_005582.1:p.Arg572Ter	3
**MRE11A**	stop_gained	NM_005591.3:c.1726C>T	NP_005582.1:p.Arg576Ter	2
**MRE11A**	splice_acceptor_variant	NM_005591.3:c.1927-2A>G	.	1

**MRE11A**	splice_acceptor_variant	NM_005591.3:c.21-6_26del	NP_005582.1:p.?	1
**MRE11A**	frameshift_variant	NM_005591.3:c.820_821del	NP_005582.1:p.Leu274Phefs*16	1
**RAD50**	splice_acceptor_variant	NM_005732.3:c.1794-1G>T	.	1
**RAD50**	stop_gained	NM_005732.3:c.1958C>A	NP_005723.2:p.Ser653Ter	1
**RAD50**	stop_gained	NM_005732.3:c.2116C>T	NP_005723.2:p.Arg706Ter	2
**RAD50**	frameshift_variant	NM_005732.3:c.229_230del	NP_005723.2:p.Asp77Cysfs*11	1
**RAD50**	stop_gained	NM_005732.3:c.2467C>T	NP_005723.2:p.Arg823Ter	1
**RAD50**	frameshift_variant	NM_005732.3:c.2505dup	NP_005723.2:p.Gln836Thrfs*9	2
**RAD50**	frameshift_variant	NM_005732.3:c.2534dup	NP_005723.2:p.Ile846Aspfs*2	1
**RAD50**	frameshift_variant	NM_005732.3:c.2675_2676del	NP_005723.2:p.Val892Glyfs*5	1
**RAD50**	frameshift_variant	NM_005732.3:c.3209del	NP_005723.2:p.Asn1070Ilefs*6	1
**RAD50**	stop_gained	NM_005732.3:c.3229C>T	NP_005723.2:p.Arg1077Ter	1
**RAD50**	frameshift_variant	NM_005732.3:c.326_329del	NP_005723.2:p.Thr109Asnfs*20	1
**RAD50**	frameshift_variant	NM_005732.3:c.354del	NP_005723.2:p.Thr119Leufs*11	1
**RAD50**	splice_donor_variant	NM_005732.3:c.3612_3618+5del	NP_005723.2:p.?	1
**RAD50**	start_lost	NM_005732.3:c.3G>A	NP_005723.2:p.Met1Ile	3
**CHEK2**	frameshift_variant	NM_007194.3:c.1100del	NP_009125.1:p.Thr367Metfs*15	53
**CHEK2**	frameshift_variant	NM_007194.3:c.1263del	NP_009125.1:p.Ser422Valfs*15	6
**CHEK2**	splice_donor_variant	NM_007194.3:c.1375+1G>A	.	1
**CHEK2**	splice_acceptor_variant	NM_007194.3:c.1462-1G>A	.	2
**CHEK2**	missense_variant	NM_007194.3:c.349A>G	NP_009125.1:p.Arg117Gly	3
**CHEK2**	frameshift_variant	NM_007194.3:c.405del	NP_009125.1:p.Lys135Asnfs*26	1
**CHEK2**	splice_donor_variant	NM_007194.3:c.444+1G>A	.	1
**CHEK2**	frameshift_variant	NM_007194.3:c.591del	NP_009125.1:p.Val198Phefs*7	1
**CHEK2**	frameshift_variant	NM_007194.3:c.655del	NP_009125.1:p.Glu219Asnfs*16	1
**CHEK2**	stop_gained	NM_007194.3:c.733A>T	NP_009125.1:p.Lys245Ter	1
**BRCA1**	frameshift_variant	NM_007294.3:c.1053del	NP_009225.1:p.Glu352Asnfs*22	3
**BRCA1**	frameshift_variant	NM_007294.3:c.1823_1826del	NP_009225.1:p.Lys608Ilefs*3	1
**BRCA1**	frameshift_variant	NM_007294.3:c.2019del	NP_009225.1:p.Glu673Aspfs*28	1
**BRCA1**	frameshift_variant	NM_007294.3:c.252del	NP_009225.1:p.Glu85Serfs*3	1

**BRCA1**	splice_donor_variant	NM_007294.3:c.301+1G>A	.	1
**BRCA1**	frameshift_variant	NM_007294.3:c.3329del	NP_009225.1:p.Lys1110Serfs*7	1
**BRCA1**	frameshift_variant	NM_007294.3:c.3756_3759del	NP_009225.1:p.Ser1253Argfs*10	1
**BRCA1**	missense_variant	NM_007294.3:c.5096G>A	NP_009225.1:p.Arg1699Gln	1
**BRCA1**	stop_gained	NM_007294.3:c.5251C>T	NP_009225.1:p.Arg1751Ter	1
**BRCA1**	missense_variant	NM_007294.3:c.5324T>A	NP_009225.1:p.Met1775Lys	1
**BRCA1**	missense_variant	NM_007294.3:c.53T>C	NP_009225.1:p.Met18Thr	1
**BRCA1**	stop_gained	NM_007294.3:c.664A>T	NP_009225.1:p.Lys222Ter	1
**BRCA1**	frameshift_variant	NM_007294.3:c.68_69del	NP_009225.1:p.Glu23Valfs*17	1
**BRCA1**	frameshift_variant	NM_007294.3:c.798_799del	NP_009225.1:p.Ser267Lysfs*19	1
**BRCA1**	stop_gained	NM_007294.3:c.962G>A	NP_009225.1:p.Trp321Ter	1
**FANCM**	frameshift_variant	NM_020937.2:c.1202del	NP_065988.1:p.Asn401Metfs*10	1
**FANCM**	frameshift_variant	NM_020937.2:c.1542dup	NP_065988.1:p.Lys515Glufs*20	1
**FANCM**	frameshift_variant	NM_020937.2:c.1724del	NP_065988.1:p.Gly575Valfs*11	2
**FANCM**	frameshift_variant	NM_020937.2:c.1946del	NP_065988.1:p.Pro649Glnfs*21	1
**FANCM**	stop_gained	NM_020937.2:c.2260C>T	NP_065988.1:p.Arg754Ter	1
**FANCM**	frameshift_variant	NM_020937.2:c.244del	NP_065988.1:p.Leu82Cysfs*66	3
**FANCM**	frameshift_variant	NM_020937.2:c.2568del	NP_065988.1:p.Val857Cysfs*5	1
**FANCM**	frameshift_variant	NM_020937.2:c.3589del	NP_065988.1:p.Asp1197Metfs*18	1
**FANCM**	frameshift_variant	NM_020937.2:c.3843dup	NP_065988.1:p.Pro1282Thrfs*5	1
**FANCM**	stop_gained	NM_020937.2:c.3975T>G	NP_065988.1:p.Tyr1325Ter	1
**FANCM**	stop_gained	NM_020937.2:c.403G>T	NP_065988.1:p.Gly135Ter	1
**FANCM**	frameshift_variant	NM_020937.2:c.4060_4064del	NP_065988.1:p.Arg1354Glyfs*4	1
**FANCM**	stop_gained	NM_020937.2:c.4194T>G	NP_065988.1:p.Tyr1398Ter	1
**FANCM**	frameshift_variant	NM_020937.2:c.4285del	NP_065988.1:p.Arg1429Glufs*7	1
**FANCM**	stop_gained	NM_020937.2:c.4438C>T	NP_065988.1:p.Gln1480Ter	1
**FANCM**	frameshift_variant	NM_020937.2:c.4755_4756del	NP_065988.1:p.His1585Glnfs*11	1
**FANCM**	frameshift_variant	NM_020937.2:c.502del	NP_065988.1:p.Met168Terfs	1
**FANCM**	stop_gained	NM_020937.2:c.5101C>T	NP_065988.1:p.Gln1701Ter	17
**PALB2**	frameshift_variant	NM_024675.3:c.1317del	NP_078951.2:p.Phe440Leufs*12	1

**PALB2**	frameshift_variant	NM_024675.3:c.1434_1435del	NP_078951.2:p.Gln479Glufs*7	1
**PALB2**	frameshift_variant	NM_024675.3:c.2052del	NP_078951.2:p.Arg686Glyfs*23	1
**PALB2**	frameshift_variant	NM_024675.3:c.2325dup	NP_078951.2:p.Phe776Ilefs*26	1
**PALB2**	frameshift_variant	NM_024675.3:c.2727_2728del	NP_078951.2:p.Thr911Leufs*16	1
**PALB2**	frameshift_variant	NM_024675.3:c.3026del	NP_078951.2:p.Pro1009Leufs*6	1
**PALB2**	frameshift_variant	NM_024675.3:c.304del	NP_078951.2:p.Val102Leufs*75	1
**PALB2**	stop_gained	NM_024675.3:c.3113G>A	NP_078951.2:p.Trp1038Ter	8
**PALB2**	frameshift_variant	NM_024675.3:c.509_510del	NP_078951.2:p.Arg170Ilefs*14	1
**PALB2**	frameshift_variant	NM_024675.3:c.886del	NP_078951.2:p.Met296Terfs	6
**BRIP1**	frameshift_variant	NM_032043.2:c.1234_1235del	NP_114432.2:p.Glu412Serfs*9	1
**BRIP1**	frameshift_variant	NM_032043.2:c.13del	NP_114432.2:p.Trp5Glyfs*9	1
**BRIP1**	frameshift_variant	NM_032043.2:c.1703del	NP_114432.2:p.Asn568Metfs*22	1
**BRIP1**	frameshift_variant	NM_032043.2:c.1727del	NP_114432.2:p.Asn576Ilefs*14	1
**BRIP1**	frameshift_variant	NM_032043.2:c.2012del	NP_114432.2:p.Glu671Glyfs*17	1
**BRIP1**	stop_gained	NM_032043.2:c.2400C>G	NP_114432.2:p.Tyr800Ter	1
**BRIP1**	frameshift_variant	NM_032043.2:c.2684_2687del	NP_114432.2:p.Ser895Terfs	1
**BRIP1**	stop_gained	NM_032043.2:c.2765T>G	NP_114432.2:p.Leu922Ter	2
**BRIP1**	frameshift_variant	NM_032043.2:c.2798_2799del	NP_114432.2:p.Asn933Ilefs*5	3
**BRIP1**	frameshift_variant	NM_032043.2:c.2798del	NP_114432.2:p.Asn933Ilefs*52	8
**BRIP1**	splice_acceptor_variant	NM_032043.2:c.2906-2A>C	.	1
**BRIP1**	frameshift_variant	NM_032043.2:c.2990_2993del	NP_114432.2:p.Thr997Argfs*61	1
**BRIP1**	stop_gained	NM_032043.2:c.3310G>T	NP_114432.2:p.Glu1104Ter	1
**RAD51C**	splice_region_variant	NM_058216.2:c.1026+5_1026+7del	.	1
**RAD51C**	stop_gained	NM_058216.2:c.397C>T	NP_478123.1:p.Gln133Ter	1
**RAD51C**	frameshift_variant	NM_058216.2:c.557_558dup	NP_478123.1:p.His187Asnfs*53	1
**RAD51C**	stop_gained	NM_058216.2:c.577C>T	NP_478123.1:p.Arg193Ter	1
**RAD51C**	frameshift_variant	NM_058216.2:c.732del	NP_478123.1:p.Ile244Metfs*9	1
**RAD51C**	missense_variant	NM_058216.2:c.773G>A	NP_478123.1:p.Arg258His	1
**RAD51C**	frameshift_variant	NM_058216.2:c.774del	NP_478123.1:p.Thr259Leufs*4	1
**RAD51C**	stop_gained	NM_058216.2:c.955C>T	NP_478123.1:p.Arg319Ter	1

**RAD51C**	stop_gained	NM_058216.2:c.97C>T	NP_478123.1:p.Gln33Ter	1

[1]Pathogenic (including likely pathogenic) as defined by ClinVar and protein truncating variants that are absent from ClinVar (accessed July 2020). Excludes carriers of a protein truncating variants located in the last coding exon.

Supplementary Table 4

Pathogenic[1] variant carriers identified by gene-panel testing and the odds ratios (ORs) and corresponding 95% confidence intervals (CIs) for associations with overall breast cancer.

Gene	Number of carriers of pathogenic variants who are Cases	Controls	Number of non-carriers of pathogenic variants who are Cases	Controls	Unadjusted* OR (95% CI)	p	Adjusted for age only OR (95% CI)	p	Adjusted** OR (95% CI)	p	
ATM	17	25	1447	7386	3.5 (1.8-6.4)	0.0002	3.9 (1.6-9.3)	0.004	3.4 (1.4-8.4)	0.009	**
BARD1	3	3	1461	7408	5.1 (0.94-27.4)	0.06	6.9 (0.61-71.7)	0.1	8.2 (0.73-83)	0.09	.
BRCA1	46	6	1418	7405	40 (18.5-105)	<0.0001	5.8 (2.4-18)	<0.0001	5.3 (2.1-16.2)	0.0001	***
BRCA2	43	21	1421	7390	10.6 (6.4-18.3)	<0.0001	4.2 (2-9.3)	0.0001	4 (1.9-9.1)	0.0002	***
BRIP1	8	13	1456	7398	3.1 (1.2-7.4)	0.02	2.7 (0.7-9.4)	0.2	2.8 (0.77-9.9)	0.1	
CDH1	1	0	1463	7411	-	-	-	-	-	-	
CHEK2	19	35	1445	7376	2.8 (1.6-4.8)	0.0009	1.4 (0.63-3.3)	0.4	1.3 (0.53-3)	0.6	
FANCM	3	19	1461	7392	0.8 (0.19-2.3)	0.7	0.97 (0.16-4.8)	1	0.8 (0.12-4.2)	0.8	
MLH1	0	0	1464	7411	-	-	-	-	-	-	
MRE11A	0	5	1464	7406	-	-	-	-	-	-	
MSH2	0	1	1464	7410	-	-	-	-	-	-	
MSH6	3	3	1461	7408	5.1 (0.94-27.4)	0.06	4.8 (0.5-41.6)	0.2	4.7 (0.52-41.3)	0.2	
MUTYH***	0	0	1464	7411	-	-	-	-	-	-	
NBN	1	11	1463	7400	0.46 (0.03-2.4)	0.4	1.7 (0.08-12.3)	0.7	2 (0.09-14.3)	0.6	
NF1	2	4	1462	7407	2.5 (0.35-13)	0.3	4.8 (0.39-39.9)	0.2	4.7 (0.4-40)	0.2	
PALB2	7	10	1457	7401	3.6 (1.3-9.3)	0.02	4.9 (1.3-18.7)	0.02	4.3 (1-17)	0.04	*
PMS2	0	0	1464	7411	-	-	-	-	-	-	
PTEN	0	1	1464	7410	-	-	-	-	-	-	
RAD50	2	11	1462	7400	0.92 (0.14-3.4)	0.9	0.33 (0.04-2.2)	0.3	0.3 (0.04-2)	0.2	
RAD51C	0	4	1464	7407	-	-	-	-	-	-	
RAD51D	1	4	1463	7407	1.3 (0.06-8.6)	0.8	0.27 (0.01-5.6)	0.4	0.25 (0.01-5.5)	0.4	
STK11	0	0	1464	7411	-	-	-	-	-	-	
TP53	6	1	1458	7410	30.5 (5.2-576)	<0.0001	15.4 (0.72-856)	0.1	19.9 (0.9-1125)	0.06	.

[1] Pathogenic (including likely pathogenic) as defined by ClinVar and protein truncating variants that are absent from ClinVar (accessed July 2020).

Excludes carriers of a protein truncating variants located in the last coding exon.

[*]Unadjusted ORs are biased by ascertainment (and included here for completeness), but unadjusted p-values are valid.

[**] Adjusted for age, height, body mass index, number of children, number of years of education and number of alcoholic drinks per day.

[***] This study excludes mono-allelic carriers of *MUTYH* pathogenic variants.

Supplementary Table 5

Pathogenic[1] variant carriers identified by gene-panel testing and the odds ratios (ORs) and corresponding 95% confidence intervals (CIs) for associations with ER-negative breast cancer.

	Number of carriers of pathogenic variants who are		Number of non-carriers of pathogenic variants who are		Unadjusted*		Adjusted for age only		Adjusted**		
Gene	Cases	Controls	Cases	Controls	OR (95% CI)	p	OR (95% CI)	p	OR (95% CI)	p	
ATM	1	41	448	8385	0.46 (0.03-2.1)	0.38	0.22 (0.01-1.1)	0.077	0.2 (0.01-1)	0.058	.
BARD1	1	5	448	8421	3.8 (0.2-23.4)	0.3	2.8 (0.13-26.3)	0.44	2.6 (0.12-25.9)	0.46	
BRCA1	39	13	410	8413	61.6 (33.5-121)	8.80E-41	15.1 (7.8-31.5)	1.30E-17	15.8 (7.9-33.9)	7.00E-17	***
BRCA2	13	51	436	8375	4.9 (2.5-8.8)	1.70E-05	1.4 (0.68-2.6)	0.36	1.4 (0.68-2.6)	0.37	
BRIP1	3	18	446	8408	3.1 (0.73-9.3)	0.11	1.9 (0.37-7.2)	0.42	2 (0.4-7.5)	0.37	
CDH1	0	1	449	8425	-	-	-	-	-	-	
CHEK2	2	52	447	8374	0.72 (0.12-2.3)	0.63	0.28 (0.04-0.98)	0.046	0.26 (0.04-0.94)	0.039	*
FANCM	1	21	448	8405	0.89 (0.05-4.3)	0.91	1.1 (0.06-7.5)	0.91	1 (0.05-7)	0.97	
MLH1	0	0	449	8426	-	-	-	-	-	-	
MRE11A	0	5	449	8421	-	-	-	-	-	-	
MSH2	0	1	449	8425	-	-	-	-	-	-	
MSH6	1	5	448	8421	3.8 (0.2-23.4)	0.3	2.4 (0.11-22.3)	0.51	2.3 (0.1-21.1)	0.52	
MUTYH***	3	57	380	8096	1.1 (0.27-3)	0.85	0.98 (0.22-3.1)	0.97	1.1 (0.24-3.3)	0.94	
NBN	0	12	449	8414	-	-	-	-	-	-	
NF1	1	5	448	8421	3.8 (0.2-23.4)	0.3	6.1 (0.25-61.4)	0.22	5.9 (0.24-60)	0.23	
PALB2	0	17	449	8409	-	-	-	-	-	-	
PMS2	0	0	449	8426	-	-	-	-	-	-	
PTEN	0	1	449	8425	-	-	-	-	-	-	
RAD50	0	13	449	8413	-	-	-	-	-	-	
RAD51C	0	4	449	8422	-	-	-	-	-	-	
RAD51D	1	4	448	8422	4.7 (0.24-31.8)	0.24	2.4 (0.1-41.4)	0.53	2.6 (0.1-46.7)	0.52	
STK11	0	0	449	8426	-	-	-	-	-	-	
TP53	3	4	446	8422	14.2 (2.8-64.4)	0.0031	2.6 (0.46-14.5)	0.26	2.9 (0.5-16.7)	0.22	

[1] Pathogenic (including likely pathogenic) as defined by ClinVar and protein truncating variants that are absent from ClinVar (accessed July 2020).

Excludes carriers of a protein truncating variants located in the last coding exon.

*Unadjusted ORs are biased by ascertainment (and included here for completeness), but unadjusted p-values are valid.

** Adjusted for age, height, body mass index, number of children, number of years of education and number of alcoholic drinks per day.

*** This study excludes mono-allelic carriers of *MUTYH* pathogenic variants.

Supplementary Table 6

Pathogenic[1] variant carriers identified by gene-panel testing and the odds ratios (ORs) and corresponding 95% confidence intervals (CIs) for associations with ER-positive breast cancer.

	Number of carriers of pathogenic variants who are		Number of non-carriers of pathogenic variants who are		Unadjusted*		Adjusted for age only		Adjusted**		
Gene	Cases	Controls	Cases	Controls	OR (95% CI)	p	OR (95% CI)	p	OR (95% CI)	p	
ATM	14	28	823	8010	4.9 (2.5-9.1)	1.90E-05	4.9 (2.1-10.8)	0.00027	4.5 (2-10)	0.00055	***
BARD1	1	5	836	8033	1.9 (0.1-11.9)	0.58	1.2 (0.06-10.5)	0.89	1.3 (0.06-11.9)	0.83	
BRCA1	6	46	831	7992	1.3 (0.48-2.7)	0.61	0.19 (0.07-0.43)	1.10E-05	0.17 (0.06-0.4)	6.00E-06	***
BRCA2	28	36	809	8002	7.7 (4.6-12.6)	4.80E-13	2.6 (1.4-4.7)	0.0015	2.6 (1.5-4.7)	0.0012	**
BRIP1	4	17	833	8021	2.3 (0.65-6.1)	0.18	1.3 (0.32-4.6)	0.68	1.5 (0.38-5.1)	0.52	
CDH1	1	0	836	8038	-	-	-	-	-	-	
CHEK2	15	39	822	7999	3.7 (2-6.7)	0.00011	2.3 (1.1-4.8)	0.029	1.9 (0.88-4.2)	0.1	
FANCM	2	20	835	8018	0.96 (0.15-3.3)	0.96	1.2 (0.17-5.6)	0.82	1.1 (0.15-5.1)	0.93	
MLH1	0	0	837	8038	-	-	-	-	-	-	
MRE11A	0	5	837	8033	-	-	-	-	-	-	
MSH2	0	1	837	8037	-	-	-	-	-	-	
MSH6	2	4	835	8034	4.8 (0.67-24.7)	0.11	3.5 (0.38-25.6)	0.24	3.6 (0.4-25.4)	0.23	
MUTYH***	9	51	742	7734	1.8 (0.84-3.6)	0.12	1.9 (0.79-4.4)	0.14	1.9 (0.77-4.5)	0.15	
NBN	1	11	836	8027	0.87 (0.05-4.5)	0.89	2.8 (0.14-17.1)	0.41	3.2 (0.16-19.2)	0.36	
NF1	1	5	836	8033	1.9 (0.1-11.9)	0.58	2.4 (0.1-21.2)	0.52	2.5 (0.11-21.8)	0.49	
PALB2	7	10	830	8028	6.8 (2.5-17.7)	0.00052	9.8 (2.9-31.9)	0.00037	8.8 (2.5-29.1)	0.0012	**
PMS2	0	0	837	8038	-	-	-	-	-	-	
PTEN	0	1	837	8037	-	-	-	-	-	-	
RAD50	2	11	835	8027	1.7 (0.27-6.5)	0.5	1.1 (0.15-6)	0.88	1.1 (0.14-5.7)	0.92	
RAD51C	0	4	837	8034	-	-	-	-	-	-	
RAD51D	0	5	837	8033	-	-	-	-	-	-	
STK11	0	0	837	8038	-	-	-	-	-	-	
TP53	2	5	835	8033	3.8 (0.55-17.9)	0.15	0.6 (0.08-3.2)	0.56	0.67 (0.09-3.6)	0.65	

[1] Pathogenic (including likely pathogenic) as defined by ClinVar and protein truncating variants that are absent from ClinVar (accessed July 2020).

Excludes carriers of a protein truncating variants located in the last coding exon.

*Unadjusted ORs are biased by ascertainment (and included here for completeness), but unadjusted p-values are valid.

** Adjusted for age, height, body mass index, number of children, number of years of education and number of alcoholic drinks per day.

*** This study excludes mono-allelic carriers of *MUTYH* pathogenic variants.

Supplementary Table 7

A sensitivity analysis that included only ABCFS participants (ASPREE participants were excluded)

Gene	Number of carriers of pathogenic variants who are Cases	Controls	Number of non-carriers of pathogenic variants who are Cases	Controls	Unadjusted* OR (95% CI)	p	Adjusted for age only OR (95% CI)	p	Adjusted** OR (95% CI)	p	
ATM	17	3	1447	859	3.4 (1.1-14.4)	0.028	3.3 (1.1-14.4)	0.029	3.1 (1.1-13.5)	0.04	*
BARD1	3	0	1461	862	-	-	-	-	-	-	
BRCA1	46	4	1418	858	7 (2.8-23.1)	1.60E-06	7.2 (2.9-23.9)	1.00E-06	7.1 (2.8-23.8)	1.80E-06	***
BRCA2	43	6	1421	856	4.3 (2-11.3)	7.90E-05	4.4 (2-11.6)	6.50E-05	4.4 (2-11.6)	6.70E-05	***
BRIP1	8	2	1456	860	2.4 (0.59-15.7)	0.24	2.4 (0.59-15.7)	0.24	2.6 (0.65-17.5)	0.19	
CDH1	1	0	1463	862	-	-	-	-	-	-	
CHEK2	19	7	1445	855	1.6 (0.7-4.1)	0.27	1.6 (0.71-4.2)	0.26	1.5 (0.64-3.8)	0.38	
FANCM	3	2	1461	860	0.88 (0.15-6.7)	0.89	0.88 (0.15-6.7)	0.89	0.8 (0.13-6.1)	0.81	
MLH1	0	0	1464	862	-	-	-	-	-	-	
MRE11A	0	0	1464	862	-	-	-	-	-	-	
MSH2	0	1	1464	861	-	-	-	-	-	-	
MSH6	3	0	1461	862	-	-	-	-	-	-	
MUTYH***	13	7	1289	823	1.2 (0.48-3.2)	0.72	1.2 (0.47-3.1)	0.75	1.2 (0.5-3.3)	0.65	
NBN	1	0	1463	862	-	-	-	-	-	-	
NF1	2	0	1462	862	-	-	-	-	-	-	
PALB2	7	1	1457	861	4.1 (0.73-77.4)	0.12	4.1 (0.72-76.2)	0.12	3.8 (0.65-72.4)	0.15	
PMS2	0	0	1464	862	-	-	-	-	-	-	
PTEN	0	0	1464	862	-	-	-	-	-	-	
RAD50	2	4	1462	858	0.29 (0.04-1.5)	0.14	0.29 (0.04-1.5)	0.14	0.28 (0.04-1.4)	0.12	
RAD51C	0	1	1464	861	-	-	-	-	-	-	
RAD51D	1	1	1463	861	0.59 (0.02-14.9)	0.71	0.61 (0.02-15.4)	0.73	0.58 (0.02-14.7)	0.7	
STK11	0	0	1464	862	-	-	-	-	-	-	
TP53	6	0	1458	862	-	-	-	-	-	-	

*Unadjusted ORs are biased by ascertainment (and included here for completeness), but unadjusted p-values are valid.

** Adjusted for age, height, body mass index, number of children, number of years of education and number of alcoholic drinks per day.

*** This study excludes mono-allelic carriers of *MUTYH* pathogenic variants.

Supplementary Table 8

A sensitivity analysis where a small number of women with pathogenic variants in multiple genes were included

Gene	Number of carriers of pathogenic variants who are Cases	Controls	Number of non-carriers of pathogenic variants who are Cases	Controls	Unadjusted* OR (95% CI)	p	Adjusted for age only OR (95% CI)	p	Adjusted** OR (95% CI)	p	
**ATM**	17	26	1454	7389	3.3 (1.8-6.1)	0.00032	3.8 (1.6-9.1)	0.0038	3.4 (1.4-8.3)	0.0089	**
**BARD1**	3	3	1468	7412	5 (0.93-27.3)	0.059	6.8 (0.6-71.6)	0.12	8.2 (0.72-82.9)	0.091	.
**BRCA1**	51	7	1420	7408	38 (18.4-92)	2.50E-33	6.3 (2.6-18.6)	6.70E-06	5.9 (2.4-17.2)	1.90E-05	***
**BRCA2**	45	23	1426	7392	10.1 (6.2-17.1)	4.70E-20	4.1 (2-9)	8.70E-05	4 (1.9-8.9)	0.00015	***
**BRIP1**	9	13	1462	7402	3.5 (1.4-8.1)	0.0068	3 (0.83-10)	0.094	3.1 (0.88-10.3)	0.079	.
**CDH1**	1	0	1470	7415	-	-	-	-	-	-	
**CHEK2**	20	36	1451	7379	2.8 (1.6-4.8)	0.00051	1.5 (0.69-3.5)	0.3	1.4 (0.6-3.4)	0.43	
**FANCM**	5	19	1466	7396	1.3 (0.44-3.3)	0.58	1.7 (0.38-6.5)	0.48	1.6 (0.36-6.2)	0.53	
**MLH1**	0	0	1471	7415	-	-	-	-	-	-	
**MRE11A**	0	5	1471	7410	-	-	-	-	-	-	
**MSH2**	0	1	1471	7414	-	-	-	-	-	-	
**MSH6**	3	3	1468	7412	5 (0.93-27.3)	0.059	4.8 (0.5-41.5)	0.17	4.7 (0.52-41.3)	0.16	
**MUTYH*** **	17	48	1454	7367	1.8 (1-3.1)	0.05	1.9 (0.85-4.2)	0.11	2 (0.88-4.4)	0.096	.
**NBN**	1	11	1470	7404	0.46 (0.03-2.4)	0.4	1.7 (0.08-12.3)	0.68	2 (0.09-14.3)	0.6	
**NF1**	2	4	1469	7411	2.5 (0.35-12.9)	0.32	4.8 (0.39-39.8)	0.21	4.7 (0.4-39.9)	0.21	
**PALB2**	9	11	1462	7404	4.1 (1.7-10)	0.0029	5.6 (1.7-19.1)	0.0056	4.9 (1.4-17.7)	0.014	*
**PMS2**	0	0	1471	7415	-	-	-	-	-	-	
**PTEN**	0	1	1471	7414	-	-	-	-	-	-	
**RAD50**	2	12	1469	7403	0.84 (0.13-3.1)	0.82	0.33 (0.04-2.1)	0.25	0.3 (0.03-2)	0.21	
**RAD51C**	0	4	1471	7411	-	-	-	-	-	-	
**RAD51D**	2	5	1469	7410	2 (0.29-9.4)	0.43	0.56 (0.05-8)	0.64	0.49 (0.05-7.2)	0.57	
**STK11**	0	0	1471	7415	-	-	-	-	-	-	
**TP53**	6	1	1465	7414	30.4 (5.2-574)	5.60E-05	15.4 (0.71-856)	0.096	19.8 (0.9-1123)	0.063	.

*Unadjusted ORs are biased by ascertainment (and included here for completeness), but unadjusted p-values are valid.

** Adjusted for age, height, body mass index, number of children, number of years of education and number of alcoholic drinks per day.

*** This study excludes mono-allelic carriers of *MUTYH* pathogenic variants.
