# Supplementary material for: Population-based estimates of breast cancer risk for carriers of pathogenic variants identified by gene-panel testing
Source: NPJ Breast Cancer. 2021 Dec 9;7:153. doi: 10.1038/s41523-021-00360-3 (PMC8660783; doi:10.1038/s41523-021-00360-3)
Supplement: Supplementary file 2 — Reporting Summary [file 41523_2021_360_MOESM2_ESM.pdf]

nature portfolio

Corresponding author(s): Melissa Southey

Last updated by author(s): Aug 19, 2021

Reporting Summary

Nature Portfolio wishes to improve the reproducibility of the work that we publish. This form provides structure for consistency and transparency in reporting. For further information on Nature Portfolio policies, see our Editorial Policies and the Editorial Policy Checklist.

Statistics

For all statistical analyses, confirm that the following items are present in the figure legend, table legend, main text, or Methods section.

n/a	Confirmed	
☐	☒	The exact sample size (*n*) for each experimental group/condition, given as a discrete number and unit of measurement
☒	☐	A statement on whether measurements were taken from distinct samples or whether the same sample was measured repeatedly
☐	☒	The statistical test(s) used AND whether they are one- or two-sided<br>*Only common tests should be described solely by name; describe more complex techniques in the Methods section.*
☐	☒	A description of all covariates tested
☐	☒	A description of any assumptions or corrections, such as tests of normality and adjustment for multiple comparisons
☐	☒	A full description of the statistical parameters including central tendency (e.g. means) or other basic estimates (e.g. regression coefficient) AND variation (e.g. standard deviation) or associated estimates of uncertainty (e.g. confidence intervals)
☐	☒	For null hypothesis testing, the test statistic (e.g. *F*, *t*, *r*) with confidence intervals, effect sizes, degrees of freedom and *P* value noted<br>*Give P values as exact values whenever suitable.*
☒	☐	For Bayesian analysis, information on the choice of priors and Markov chain Monte Carlo settings
☒	☐	For hierarchical and complex designs, identification of the appropriate level for tests and full reporting of outcomes
☐	☒	Estimates of effect sizes (e.g. Cohen's *d*, Pearson's *r*), indicating how they were calculated

Our web collection on statistics for biologists contains articles on many of the points above.

Software and code

Policy information about availability of computer code

Data collection

Provide a description of all commercial, open source and custom code used to collect the data in this study, specifying the version used OR state that no software was used.

Data analysis

Raw sequencing reads were mapped to the reference genome (GRCh37) using bwa-mem 0.7.17.
Target coverage was calculated using bedtools.
Variants were called using VarDict 1.7 and annotated using VarSeq VSClinical v2.2.
Statistical analyses were performed using R v.3.4.2

For manuscripts utilizing custom algorithms or software that are central to the research but not yet described in published literature, software must be made available to editors and reviewers. We strongly encourage code deposition in a community repository (e.g. GitHub). See the Nature Portfolio guidelines for submitting code & software for further information.

Data

Policy information about availability of data

All manuscripts must include a data availability statement. This statement should provide the following information, where applicable:

- Accession codes, unique identifiers, or web links for publicly available datasets
- A description of any restrictions on data availability
- For clinical datasets or third party data, please ensure that the statement adheres to our policy

All rare pathogenic variants identified are reported in the Supplementary Tables.
Other data is available upon request to the corresponding author (for data related to ABCFS) and paul.lacaze@monash.edu (for data related to ASPREE).

Field-specific reporting

Please select the one below that is the best fit for your research. If you are not sure, read the appropriate sections before making your selection.

☒ Life sciences ☐ Behavioural & social sciences ☐ Ecological, evolutionary & environmental sciences

For a reference copy of the document with all sections, see nature.com/documents/nr-reporting-summary-flat.pdf

Life sciences study design

All studies must disclose on these points even when the disclosure is negative.

Sample size	1,464 women diagnosed with breast cancer and 862 age-matched controls participating in the ABCFS<br>6,549 women from the APSREE study
Data exclusions	Male participants and female participants to ASPREE who reported a personal of family history of breast cancer at baseline were excluded from the statistical analyses.<br>Participants for whom sequencing yielded less than 80% of the target covered at 30X were excluded from the statistical analyses.<br>Carriers of more than one variant were excluded from the main analysis (but included in sensitivity analysis Supp Table 8).<br>Mono-allelic carriers of MUTYH pathogenic variants were excluded from the statistical analyses (due to insufficient evidence for association with breast cancer beyon bi-allelic carriers).
Replication	No replication analyses were conducted. However two sensitivity analyses were performed:<br>- Supp Table 7: a sensitivity analysis that included only ABCFS participants (excluded ASPREE participants)<br>- Supp Table 8: a sensitivity analysis that included the small number of women with pathogenic variants in more than one gene.
Randomization	This is a case-control study.
Blinding	Investigators were blinded to the clinical characteristics of the participants during the generation of the genomic dataset.

Reporting for specific materials, systems and methods

We require information from authors about some types of materials, experimental systems and methods used in many studies. Here, indicate whether each material, system or method listed is relevant to your study. If you are not sure if a list item applies to your research, read the appropriate section before selecting a response.

Materials & experimental systems

n/a	Involved in the study	
☒	☐	Antibodies
☒	☐	Eukaryotic cell lines
☒	☐	Palaeontology and archaeology
☒	☐	Animals and other organisms
☐	☒	Human research participants
☐	☒	Clinical data
☒	☐	Dual use research of concern

Methods

n/a	Involved in the study	
☒	☐	ChIP-seq
☒	☐	Flow cytometry
☒	☐	MRI-based neuroimaging

Human research participants

Policy information about studies involving human research participants

Population characteristics	Age at diagnosis of breast cancer, age at enrolment, height, body mass index, number of children, number of years of education and number of alcoholic drinks per day
Recruitment	ABCFS probands were either breast cancer cases (identified through population-complete cancer registries) or age-matched controls (identified through Electoral rolls). ASPREE study is a randomized, placebo-controlled study of low-dose aspirin.
Ethics oversight	Monash University and Melbourne University.

Note that full information on the approval of the study protocol must also be provided in the manuscript.

Clinical data

Policy information about clinical studies

All manuscripts should comply with the ICMJE guidelines for publication of clinical research and a completed CONSORT checklist must be included with all submissions.

Clinical trial registration	*Provide the trial registration number from ClinicalTrials.gov or an equivalent agency.*
Study protocol	*Note where the full trial protocol can be accessed OR if not available, explain why.*
Data collection	*Describe the settings and locales of data collection, noting the time periods of recruitment and data collection.*
Outcomes	*Describe how you pre-defined primary and secondary outcome measures and how you assessed these measures.*
